# Supplementary material for: Volatile DMNT directly protects plants against Plutella xylostella by disrupting the peritrophic matrix barrier in insect midgut
Source: eLife. 2021 Feb 18;10:e63938. doi: 10.7554/eLife.63938 (PMC7924945; doi:10.7554/eLife.63938)
Supplement: Supplementary file 2. [file elife-63938-supp2.doc]

**Supplementary file 2 Primers used in this study**

| **Name** | **Primer sequences (5′-3′)** | **Application** |
| --- | --- | --- |
| **PxMucin--F**  **PxMucin--R**  **dsRNA-PxMucin--F**  **dsRNA-PxMucin--R**  **dsRNA-GFP-F**  **dsRNA-GFP-R**  **PxMucin--F(RNAi)**  **PxMucin--R(RNAi)** | CTGCCTCATGAGACTGAATG  TCTCCACTGACGCTGATCTC  GGATCCTAATACGACTCACTATAGGATCACTGAAGCTCAGACTGTCG  GGATCCTAATACGACTCACTATAGGCGCAGTAGTAGAACTTGTCGCAG  GGATCCTAATACGACTCACTATAGGCAGTGCTTCAGCCGCTACCC  GGATCCTAATACGACTCACTATAGGACTCCAGCAGGACCATGTGAT  AACCTCGTCGAGCACTCTTG  GAGCTTTATGTATTTGTTCGAT | RT-PCR analysis of *PxMucin*  synthesis of dsRNA  RT-PCR for RNAi |
